# Numerical Analysis of Cavitation Erosion in 316L Steel with CrN PVD Coating

**DOI:** 10.3390/ma17174397

**Published:** 2024-09-06

**Authors:** Artur Maurin

**Affiliations:** Institute of Fluid-Flow Machinery, Polish Academy of Sciences, Hydropower Department, Fiszera 14 St., 80-231 Gdansk, Poland; amaurin@imp.gda.pl; Tel.: +48-58-5225-186

**Keywords:** PVD, cavitation erosion, ceramic coating, microjet, FEA, 316L steel, CrN

## Abstract

The erosion process of a 4 μm monolayer CrN coating deposited on 316L stainless steel due to cavitation was investigated using finite element analysis (FEA). To estimate load parameters from cavitation pit geometry resulting from high impact velocity and high strain rate, the explicit dynamic solver was employed. Water microjet impacts at velocities of 100, 200 and 500 m/s were simulated to recreate different cavitation erosion intensities observed in the experiment. The resulting damage characteristics were compared to previous studies on uncoated 316L steel. The relationship between impact velocity and postimpact geometry was examined. Simulations revealed that only impact at 500 m/s can exceed the maximum yield stress of the substrate without penetrating the coating. Subsequent impacts on the same zone deepen the impact pit and penetrate the coating, leading to direct substrate degradation. The influence of impact velocity on the coating degradation process is discussed.

## 1. Introduction

To meet constantly changing energy market demands, hydropower machinery is frequently required to operate beyond optimal efficiency conditions. There is also a trend towards increasing the specific speed of newly constructed machines to maximise power output and efficiency. Together, these factors contribute to the cavitation erosion problem. Cavitation generates pressure waves that propagate through the structure and liquid, potentially damaging the blades and channel walls in hydraulic machinery through erosion. To counter these adverse effects, the durability of materials must be increased. One of the effective solutions for this problem is ceramic coatings [1,2]. These materials provide enhanced protection against the wear and tear caused by cavitation, thereby extending the lifespan of hydropower machinery components [3].

Understanding the degradation mechanisms of ceramic coatings due to cavitation is essential to further improve these protective measures. The collapse of cavitation bubbles produces two main erosion mechanisms: microjets and shock waves. When a bubble collapses in proximity to a solid boundary, a microjet is directed towards that boundary. This occurs because the solid boundary prevents radial liquid flow beneath the bubble, resulting in minimum liquid pressure at the bubble wall near the solid surface and maximum pressure at the wall farthest from it. The fundamental physical of cavitation and bubble dynamics are well understood with the nucleation process thoroughly described in theoretical frameworks and empirical studies for both flowing and quiescent liquids [4].

From the standpoint of structural mechanics, cavitation erosion in ceramic materials occurs differently than in steel materials [5]. When the material’s fracture toughness is exceeded by the local equivalent stresses, it can lead to the introduction of cracks and the propagation of these cracks through the brittle ceramic matrix. On the microscopic level, as the stress concentrations occur, microcracks begin to form and propagate. These cracks can coalesce, leading to spallation or chipping of the ceramic.

Elastic waves (acoustic emissions) are generated by the cavitation-induced stress and travel through the ceramic structure. The magnitude and velocity of the source event determine both the energy released by the microjet impact (collision) and the amplitude of the resulting waveform. Unlike metals, which primarily undergo plastic deformation, ceramics primarily experience brittle fracture mechanisms under cavitation loads. This difference underscores the importance of improving the fracture toughness and resilience of ceramic coatings to enhance their performance in hydropower applications [3].

Damage mechanisms may differ as the thin coating of ceramic material is deposited on the metal substrate, creating a cermet. Cermets exhibit complex behaviour due to the distinct properties of their constituent materials. This complexity arises from the interplay between the metal’s ductility and toughness and the ceramic’s hardness and brittleness. This combination results in a material that can withstand harsh environments and mechanical stresses that neither constituent could endure alone [6,7,8].

Modelling and predicting the results of high-velocity collisions require a methodology that combines failure analysis, conservation laws and classical mechanics. The modelling of pitting corrosion due to the collapse of a cavitation bubble, along with the material behaviour, has been effectively simulated [9]. The damage caused by the collapse of a single cavitation bubble can be directly observed using 3D microscopes and high-speed cameras [10]. Recordings at the highest acquisition rates show that the material deforms and then relaxes to some extent, with residing substantial deformation. This entire process occurs within 2 to 3 µs [10,11].

Cavitation occurs when the local pressure in a fluid drops below the vapour pressure, leading to the formation of vapour bubbles. The exact locations and times where this pressure drop occurs can vary significantly depending on fluid dynamics, surface roughness, impurities in the fluid and other environmental factors. This randomness in bubble formation contributes to the stochastic nature of cavitation erosion. Once formed, the bubbles grow as they move to regions of higher pressure. The dynamics of bubble growth, including their size and shape, are influenced by a multitude of factors such as local pressure gradients, fluid velocity and interactions with nearby bubbles. These factors vary spatially and temporally, making the behaviour of individual bubbles difficult to predict.

Xie [12] assessed the theoretical upper limit for the velocity of a microjet impact, which is 1202.9 m/s for the collapse of a cavitation bubble with a 3.5 mm radius. However, the actual energy of the microjet dissipates significantly in the fluid, reducing this value. Many studies have attempted to determine the true velocity of microjets [9,10,11,13,14,15]. Depending on factors such as the water vapour content, the diameter of the cavitation bubble and the size of the water boundary layer, the range of impact velocity changes from 20 m/s to over 600 m/s. Investigations by Lauterborn and Bolle [15] found a maximum microjet velocity of 120 m/s, while Dular et al. [11] reported a maximum velocity of approximately 175 m/s. Wright et al. [16] observed water jet velocities in the range of 179–232 m/s. In contrast, Bourne [17], Field et al. [18] and Smith and Kinslow [19] have shown that microjet speeds can reach from 400 to 600 m/s. Consequently, for the calculations presented in this paper, the velocity range of microjet impact was chosen to be between 100 m/s and 500 m/s. Studies [20,21,22] indicate that microjets cause the maximum damage to solid surfaces when they attack at a 90-degree angle.

The study [23] simulated the plastic deformation of a microjet impact on the workpiece surface using the finite element analysis software ABAQUS. The influence of different microjet diameters was analysed. Recently [24], ABAQUS was used to conduct simulation analysis for the impact process of ultrasonic cavitation microjets on a titanium–tantalum alloy surface employing the smoothed particle hydrodynamics–finite element method (SPH–FEM), which revealed that continuous high-velocity microjet impacts produce significant cumulative material removal. Although the simulation methodology is similar, those studies did not analyse the influence of the coating on the cavitation resistance of the material.

In this paper, the degradation of 316L stainless steel coated with a 4 µm CrN monolayer was analysed using finite element (FE) structural analysis of two consecutive high-velocity microjet collisions. Section 2 presents a detailed overview of this approach. To enhance insight into the relationship between the material properties and surface deformation, Section 3 investigates the correlation between the impact velocity and postimpact pit (crater) geometry. The influence of the microjet impact on the stress distribution within the surface layer of 316L stainless steel with CrN coating is discussed in Section 4. In the experiment, the coating degradation processes include: surface microundulation in the initial stage and the formation of numerous circle-shaped craters with a penetration of about 2–3 µm, which is probably the result of high-velocity microjet impact [5]. In the simulation, only the latter mechanism was considered.

The aim of this paper was to analyse the transfer of impact energy through coating the substrate, the size of indentations and to assess the size of damaged fragments of the substrate. The obtained analysis results were compared to previous studies where the 316L steel was not protected by the coating [25]. This approach allows us to investigate the effects of different impact velocities on the coating and substrate, leading to a more comprehensive insight into the cavitation erosion process.

## 2. Materials and Methods

The 316L steel is widely utilised in hydropower applications. The considered coating substrate had a chemical composition according to the EN 10204:2004 3.1B [26] specification, presented in Table 1.

**Figure 1:**
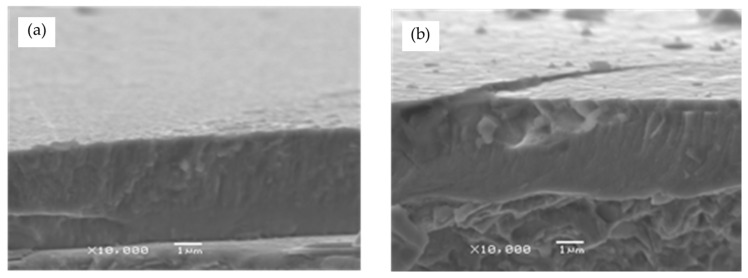
CrN coatings deposited on the 316L steel by (**a**) RMS-PVD and (**b**) CAE-PVD methods (reprinted from [5], copyright under licence no. 5818850396528 (2024), with permission from Elsevier).

To improve the 316L steel cavitation erosion resistance, CrN coatings can be applied (Figure 1). For the analysis, the reactive magnetron sputtering (RMS-PVD) CrN monolayer coating was selected as it has a significantly more uniform structure than their cathodic arc evaporation (CAE-PVD) or multilayer [27] counterparts. This approach significantly limits the number of variables and reduces uncertainties in later numerical analysis. RMS-PVD also produced at least 20 times fewer microdroplets than the CAE-PVD process. The chemical composition of the created RMS-PVD CrN coating is Cr-50.1%, N-48.5%, O-1.4%. The phase structure of the analysed coating is CrN + Cr2N + Cr(N) with a crystallite size between ~33 and ~46 nm and an Ra of 0.05–0.07 µm [5].

### 2.1. Cavitation Erosion Tests

The collapse of cavitation bubbles is a critical phase where most of the erosive damage occurs. The collapse can be highly violent, producing shock waves and high-velocity liquid jets that impact the material surface. The exact location, intensity and angle of these impacts are random, depending on how the bubble collapses, which is influenced by factors like bubble size, proximity to a surface and the surrounding pressure field. This randomness contributes to the stochastic nature of the resulting material erosion. The resulting erosion patterns on the material surface are highly irregular and unpredictable. Erosion can occur in localised spots where bubbles collapse more frequently or with greater intensity. Over time, this randomness leads to a nonuniform distribution of damage, with some areas experiencing severe erosion while others remain relatively unaffected.

**Figure 2:**
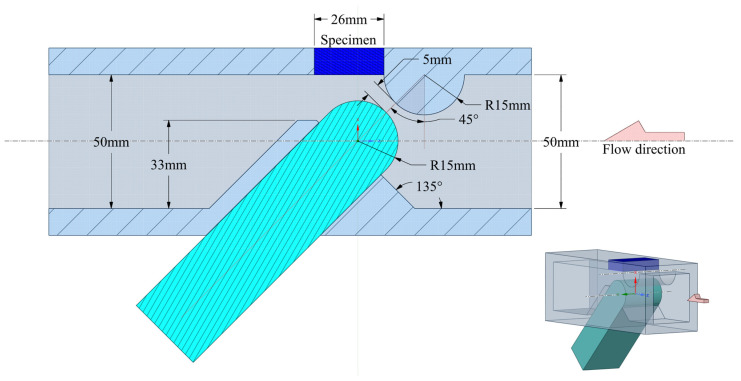
Cross-section and 3D overview of the cavitation chamber test device.

The stress response characteristics of the microjet collision process were investigated by comparing microjet postimpact geometries created in the experiment [5]. The experimental tests were performed using a cavitation chamber with a system of barricade exciters. The cross-section of the cavitation tunnel is shown in Figure 2 and described in Ref. [28]. The cavitation bubble collapse region in the cavitation chamber was located behind the barricade system near the surface of a specimen. The inlet and outlet pressures, adjusted by the inlet and outlet valves, determined the flow velocity in the cavitation chamber, which regulates the development of cavitation bubbles and cavitation intensity.

**Figure 3:**
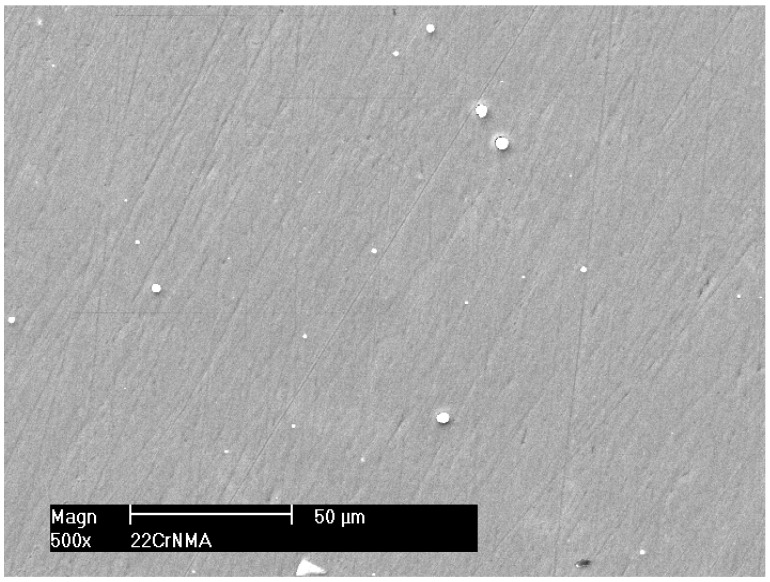
Structure of CrN coating produced by RMS-PVD method at 350 °C before erosion (reprinted from [5], copyright under licence no. 5818850396528 (2024), with permission from Elsevier).

Cavitation tests were conducted with inlet absolute pressures of 1000 kPa and 1200 kPa and outlet absolute pressures of 125 kPa and 130 kPa, respectively. The outlet pressures resulted from entirely open outlet valves. An aperture width of 5 mm was maintained between the barricades. Tap water at a temperature of 20 ± 2 °C was used as the working liquid. The initial exposure duration was 15 min, which was subsequently extended to 30, 60 and 120 min. The cavitation erosion mechanisms of the PVD coatings were analysed based on surface observations of the specimens using Philips XL-30 and SU3500, Hitachi, Tokyo, Japan, scanning electron microscopes [5]. The exemplary microscopic observation of the RMS-PVD CrN coating surface before the cavitation test is presented in Figure 3. The white speckles are the metal microdroplets as a side effect generated during coating production.

The influence of the load intensity on material degradation for ceramic coating deposited on a metal substrate is still undetermined. The resulting crater geometry corresponds to parameters such as the initial bubble size, the local collapse driving pressure gradient and the exact location of the bubble collapse. The intensity of the load depends on the number and power of the microjets and/or shock waves formed during the implosion of the cavitation bubbles [12,15,20,22].

The response of the material to cavitation-induced stresses also contributes to the stochastic nature of cavitation erosion. Different materials or even different regions within the same material may respond differently to the high-pressure impacts depending on factors like microstructure, grain boundaries and pre-existing defects. This variability adds another layer of unpredictability to the erosion process.

**Figure 4:**
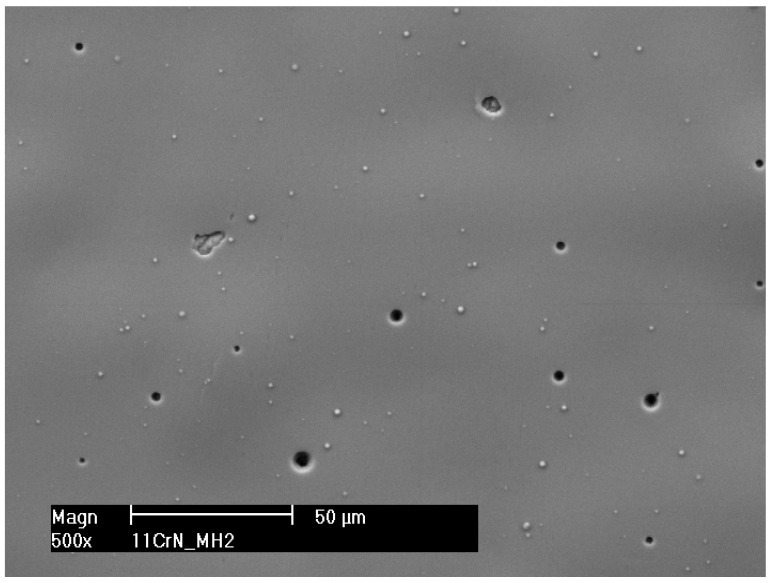
Structure of CrN coating deposited by RMS method after 1 h of erosion (reprinted from [5], copyright under licence no. 5818850396528 (2024), with permission from Elsevier).

Figure 4 represents the results after 60 min of the 1000 kPa test. Irregular surface exposition results in alterations in surface degradation. The diameter of the craters can be observed within a broad range from 2 to 5 µm. Visibility in surface undulation confirms a ductile mode of substrate damage [5]. Additionally, a void in the coating probably was initiated underneath the surface due to delamination. Typically, high shear stresses caused by high-rate impacts lead to the formation of such voids beneath the surface [29]. Comparing the surface roughness before and after the test, almost the entire coating surface experienced erosion. The results depicted in Figure 4 illustrate the inherently stochastic nature of the cavitation erosion process.

### 2.2. Numerical Model

**Figure 5:**
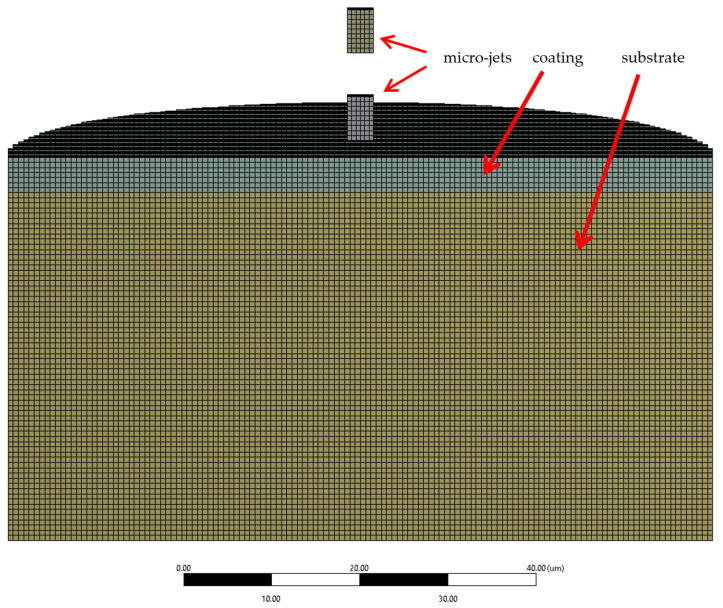
Cross section of FEA calculation domain with visible finite element mesh.

The aim of the proposed simulation method is not to reflect the real-life cavitation erosion process but to present the most probable outcome of the microjet collision event. The described approach is based on well-established material models and simulation methods. To find the microjet damage characteristics of 316L stainless steel with CrN coating subjected to a cavitation bubble collapse zone, a microjet impact was studied using a finite element analysis. The ANSYS Explicit Dynamics solver (ANSYS 2024R1) was employed. The solver uses an explicit time integration scheme, which is well suited for problems involving high-velocity collisions. This method calculates the state of a system at the next time step using only the information from the current state. The equations of motion are solved directly and explicitly for each small time increment (time step). This approach is particularly effective in dealing with the highly dynamic and nonlinear behaviour seen in high-velocity collisions where the forces and displacements change rapidly over time. Due to the explicit nature of the solver, very small time steps (1 × 10^−12^ s) are used to ensure accuracy and stability. The size of the time step is dictated by the smallest element in the mesh and the speed of the wave propagation through the material. Thus, the solver accurately models the propagation of stress waves through the material, which is a key aspect of high-velocity collisions. These waves can cause complex interactions between the coating and the substrate, such as reflections at boundaries or interfaces, which must be captured to understand the material’s response fully. The experimental verification for the used simulation method was presented and discussed in [25], where a larger set of experimental data for 316L steel without coating was available. In the current study, the scope of analysis was extended to account for the CrN coating. The cylinder-shaped specimen model had a thickness of 44 µm (including a 4 µm coating) and a diameter of 80 µm (Figure 5). The thickness of the coating was previously measured on a metallographic cross-section of the sample adopting build-in microscope measurement tools (Figure 1a) as well as using Calotte’s method (Calo test).

The solver handles contact between different bodies or within the same body dynamically. This feature is critical in high-velocity collision simulations where the interaction between different materials (e.g., coating and substrate) plays a significant role in the overall behaviour. The contact algorithms ensure that the forces, friction and potential material separation or penetration are accurately modelled. Here, the coating and the substrate contact type were defined as bonded due to strong adhesion. The side of the sample where the microjet impact occurred was designated as the free surface to allow material outflow, while the remaining two surfaces were designated as energy-absorbent impedance boundaries. By using these boundaries, we ensure that the physical effects, such as stress waves and material deformations, are not reflected back into the model, which could otherwise distort the simulation results. Instead, the energy is dispersed as it would be in a continuous material, effectively mimicking the conditions of a larger-scale model.

The accuracy of the simulation depends on the quality and resolution of the finite element mesh. In high-velocity collision scenarios, the mesh needs to be fine enough to capture the detailed response of the material, such as the formation of craters, stress concentration zones and failure initiation. To ensure symmetry of the initial conditions, a Cartesian mesh method was used with an optimal element size of 0.6 µm (Table 2), defined along the element’s edge, satisfying the Courant–Friedrichs–Lewy (CFL) condition.

For the selected mesh type, the explicit dynamics solver in ANSYS 2024R1 does not accept planar or cyclic symmetry. As a result, the model consisted of approximately 1 × 10^6^ hexahedral elements (Figure 5). The microjet size was determined based on experimental observations [30]. The microjet diameter typically measures below one tenth of the diameter of a cavitation bubble. The microjet length is assumed to be less than twice its diameter [23,24]. In this study, the diameter and length of the microjet are assumed to be 3 and 5 µm, respectively.

The theory of water hammer was used to derive numerical simulations of both the impact velocity and impact pressure [10,31,32]. The impact pressure was determined using two fundamental equations. At the moment of impact, the pressure was considered as the water hammer pressure and was calculated using the formula

(1) P=cvϱ,

where *c* is the acoustic velocity, *v* is the impact velocity and *ρ* is the density of water. Once the incompressible flow in the microjet axis reaches equilibrium, the pressure decreases to a lower value, determined by

(2) $$P=\frac{\varrho v^{2}}{2},$$

where *ρ* is water density. Because of the unpredictable nature of the transformation process between the initial and equilibrium states, the values of the impact pressure reported in the literature are diverse. In Reference [19], the impact pressure reaches about 1 GPa. According to Reference [9], the maximum impact pressure was found to be 3–4 GPa. The length of an impact depends on the material involved and ranges from 2 µs [11] to even 17 µs [13] while the rise interval is from 1 µs to 3 µs [19]. Due to their brief duration, these impacts are referred to as cavitation pulses [14]. Repeated collisions from these pulses near the solid boundary result in damage, which ultimately leads to erosion.

The calculations assume that the impact direction is perpendicular to the material, transferring the maximum amount of energy to it. Because of the constraints of the solver, temperature changes during impact were not considered. The solver includes failure criteria, allowing it to predict when and where the material will fail under the load. The erosion mechanism of the finite elements was activated in the calculations with a geometric strain limit factor set at 1.5. For simulating high-velocity collisions, this capability is essential to simulate the degradation process, including crater formation and resulting material removal. The microjet velocities selected in this study were 100, 200 and 500 m/s, which allowed for direct comparison with the results presented in [25].

In flow cavitation, the flow velocity primarily determines the number and characteristics of the cavitation bubbles. The amplitude, number of cavitation pulses and microjet impact speed rise with the flow velocity, intensifying the cavitation erosion process. Measurements of the surface hardness and the progression of damage over time underscored the importance of analysing stress development in 316L steel resulting from repeated microjet impacts [25]. Considering the cavitation erosion process, those conditions in the simulation were represented by the two successive microjet impacts.

The general material properties for coating and substrate, available in the Explicit Material library in ANSYS Engineering Data Sources, used in the simulation are detailed in Table 3. For 316L steel, the Johnson and Cook [33] the model was adapted. This model characterizes the inelastic behaviour of solids (such as elasto-plastic hardening under significant strains) as a function of the rates of equivalent plastic strain and temperature across defined ranges. In 316L steel, permanent deformation occurs when local equivalent stresses surpass the material’s yield stress (307 MPa), leading to dislocation movement, slip initiation, twinning, crack growth and phase transformations in metals [25]. When atomic planes slide past one another due to the movement of dislocations, plastic deformation (microyielding) occurs. While the accurate simulation of these phenomena is beyond the reach of any available finite element (FE) solver, choosing an appropriate set of material data (which reflect the effects of these processes) and an effective simulation strategy (which measures the energy released by microjet impacts) can provide a reasonable (volume-averaged) approximation of the cavitation erosion effects. Using volume-averaged material properties simplifies the simulation by smoothing out the effects of microstructural variations (e.g., grain boundaries, defects). This approach is suitable for capturing general trends in the material’s response to impacts, especially when the aim is to understand the overall material response rather than the behaviour of specific microstructural features. The downside is that results do not capture localised effects that could be significant in real materials such as the influence of microstructural heterogeneities on the formation of cracks or the initiation of localised plastic deformation.

The Explicit Dynamics solver incorporates complex, nonlinear material models that account for strain rate effects, plasticity, failure and other advanced material behaviours. For the CrN, the Steinberg–Guinan [34] model was used due to the model’s ability to accurately represent the material behaviour under high strain rate conditions. The Steinberg–Guinan model includes mechanisms for handling high-pressure zones, making it suitable for simulations involving extreme compressive stresses, which are present during microjet impact. In ANSYS, the Steinberg–Guinan and Johnson–Cook strength models are both used to describe the nonlinear behaviour of materials under high strain rates and/or temperature changes but they have different formulations and are used for different types of materials and applications. The comparison of the two models is presented in the following subsections.

### 2.3. Johnson–Cook Strength Model

In ANSYS, the Johnson–Cook (J–C) is a phenomenological material model used to describe the behaviour of materials under various conditions, particularly for high strain rates, high temperatures and large deformations. The strain rate correction in the Johnson–Cook material model accounts for the material’s sensitivity to the rate at which deformation occurs.

The J–C material model is expressed with the following equation for flow stress σ:

(3) $$\sigma =(A+B{\left(\varepsilon^{p}\right)}^{n})(1+Cln(\frac{{\dot{\varepsilon }}^{p}}{{\dot{\varepsilon }}_{0}}))(1-{\left(\frac{T-T_{0}}{Tm-T_{0}}\right)}^{m}),$$

where:

- A is the yield stress at reference strain rate and room temperature;
- B is the hardening modulus;
- n is the hardening exponent;
- $\varepsilon^{p}$ is the equivalent plastic strain;
- ${\dot{\varepsilon }}^{p}$ is the equivalent plastic strain rate;
- ${\dot{\varepsilon }}_{0}$ is the reference strain rate;
- T is the current temperature;
- $T_{0}$ is the reference temperature;
- Tm is the melting temperature of the material;
- m is the thermal softening coefficient.

In ANSYS, the strain rate correction term (1 + C ln (ε*^p^*/${\dot{\varepsilon }}_{0}$)) modifies the flow stress based on the rate of deformation. The parameter C represents the material’s sensitivity to the strain rate. A higher value of C indicates that the material is more sensitive to changes in strain rate.

**Figure 6:**
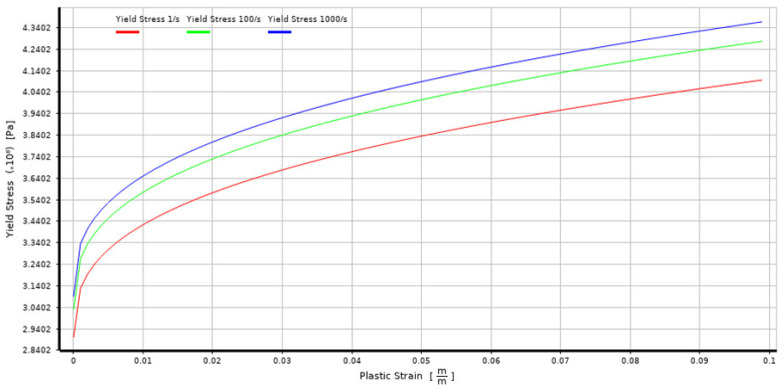
Johnson–Cook strength characteristic for 316L stainless steel.

The J–C model is widely used for metals, especially for dynamic problems involving high strain rates and temperatures such as metal cutting, impact and ballistic penetration. It is suitable for both low- and high strain rate applications. To implement the Johnson–Cook model with strain rate correction, the material properties, including the constants A, B, n, C and m, as well as the reference strain rate ${\dot{\varepsilon }}_{0}$ are taken. These parameters can be determined relatively easily from experimental data. Although the J–C model may not accurately capture complex material behaviours like phase transformations or pressure dependencies, it was selected as best fitted for the 316L substrate. The model representation of the J–C strength characteristic of 316L steel, available in the Explicit Materials library in ANSYS Engineering Data Sources, used in the simulation is presented in Figure 6.

### 2.4. Steinberg–Guinan Strength Model

The Steinberg–Guinan (S–G) model employed in calculations is designed to describe the strength of materials under high pressures, high strain rates and high temperatures, often encountered in shock physics and geophysics. The model accounts for pressure dependence of the yield strength and the effect of strain hardening and thermal softening. The yield strength $\sigma_{y}$ is typically expressed as

(4) $$\sigma_{y}=\sigma_{0}+G\left(\epsilon_{p}\right)\left[1+b\left(T-T_{0}\right)\right],$$

where:

- $\sigma_{0}$ is the initial yield strength;
- $G\left(\epsilon_{p}\right)$ is the shear modulus as a function of plastic strain;
- b is a thermal softening coefficient;
- T is the current temperature;
- $T_{0}$ is the reference temperature.

**Figure 7:**
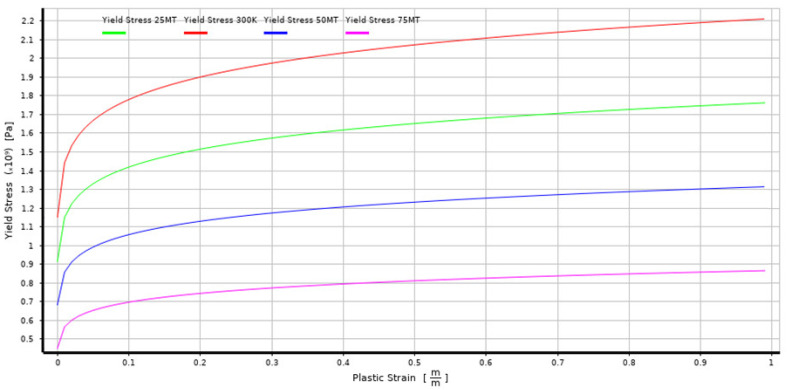
Steinberg-Guinan strength characteristic for CrN coating.

The shear modulus G itself is pressure dependent and has a functional form to capture the effects of strain hardening. The S–G model is commonly used in high-pressure physics, shock wave studies and geophysics. It is suitable for materials subjected to extreme conditions such as materials under high pressure and high strain rates. The S–G has an advantage over the J–C model for materials under high pressure. It is better suited for simulating shock wave propagation and high-pressure scenarios. However, the model parameters are more difficult to obtain and require high-pressure experimental data. The available S–G strength characteristic of the CrN coating, available in the Explicit Materials library in ANSYS Engineering Data Sources, used in the simulation is presented in Figure 7.

In the experiment, the cracks developed in the CrN coating mainly occurred from the upper to the side of undulation. Therefore, the FEM model does not account for this mechanism due to modelling limitations. In the FEM model, the phase transformation Fe-γ → Fe-α′ observed in austenitic steel substrate is simulated through the nonlinear material properties. The material properties were applied in the Ansys Engineering Data module.

In summary, by assuming the most probable impact conditions, the simulation captures the general trend of the material response, such as increased resistance to deformation or changes in the yield strength after the impacts. This approach enables a focus on the material’s general behaviour under repeated impacts without being encumbered by the complex and random variations that may arise in real-world erosion test.

## 3. Analysis Results

The stochastic nature of cavitation erosion arises from the complex, random interactions between fluid dynamics, bubble formation and collapse and material response. This randomness leads to unpredictable patterns of damage, requiring statistical approaches for modelling and predicting erosion behaviour. The proposed simulation approach does not capture the full complexity of cavitation erosion but, through established assumptions, provides valuable insights into the general behaviour of the material.

**Figure 8:**
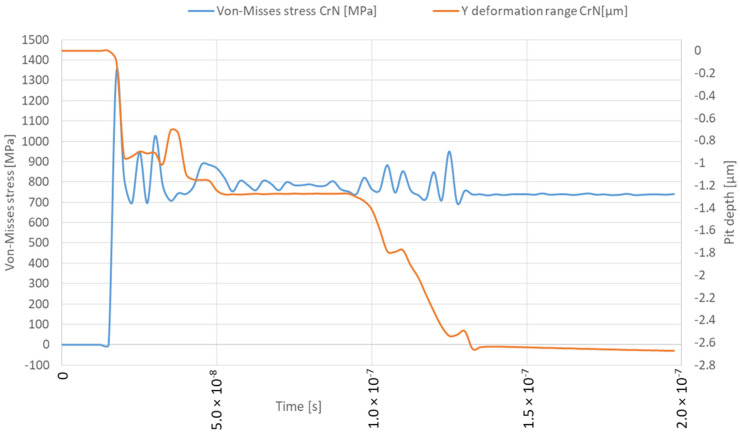
Von-Mises stresses and deformation range in the Y direction during first and second microjet collision at 100 m/s with and without protective CrN coating.

This study examined the damage caused by microjet impact erosion at three different velocities: 100, 200 and 500 m/s. The solver tracks the material deformation over time, providing a detailed representation of the crater formation process, including the material’s response to successive collisions. The established simulation model is similar to the previous studies where the 316L steel was not protected by the coating [25]. This approach allows for a direct comparison of the result with and without CrN coating. The colour map display was changed from smooth contours to band contours to emphasise the abrupt change in stress values at the contact interface between the coating and the substrate. The solver captured the transient material response (Figure 8), including the maximum stress level as well as deformation rage in the Y direction under the high strain rates associated with these collisions. The deformation range should be distinguished from the crater depth, as it can differ. The Y deformation range of the microjet collisions was obtained with the “directional deformation” tool, set up in the axis of impact for the deformed body and it primarily allows us to identify the moment of the second impact. The depth of the microjet impact was obtained using the Node Single Select tool available in ANSYS Workbench, which allows us to measure the distance when two nodes are selected together after each collision event. This approach aims to represent the transient nature of the collision, enabling the study of how different velocities affect the coating’s ability to withstand loads without penetration and how successive collisions influence coating and substrate degradation. The Von-Mises equivalent stress distributions for both the coating and substrate throughout two successive microjet collisions were calculated and represented in three snapshots, each for a different stage of the collision: the initial contact (initial stage), the midpoint of the impact duration (second stage) and the postimpact state (third stage).

### 3.1. Microjet Collision at 100 m/s

**Figure 9:**
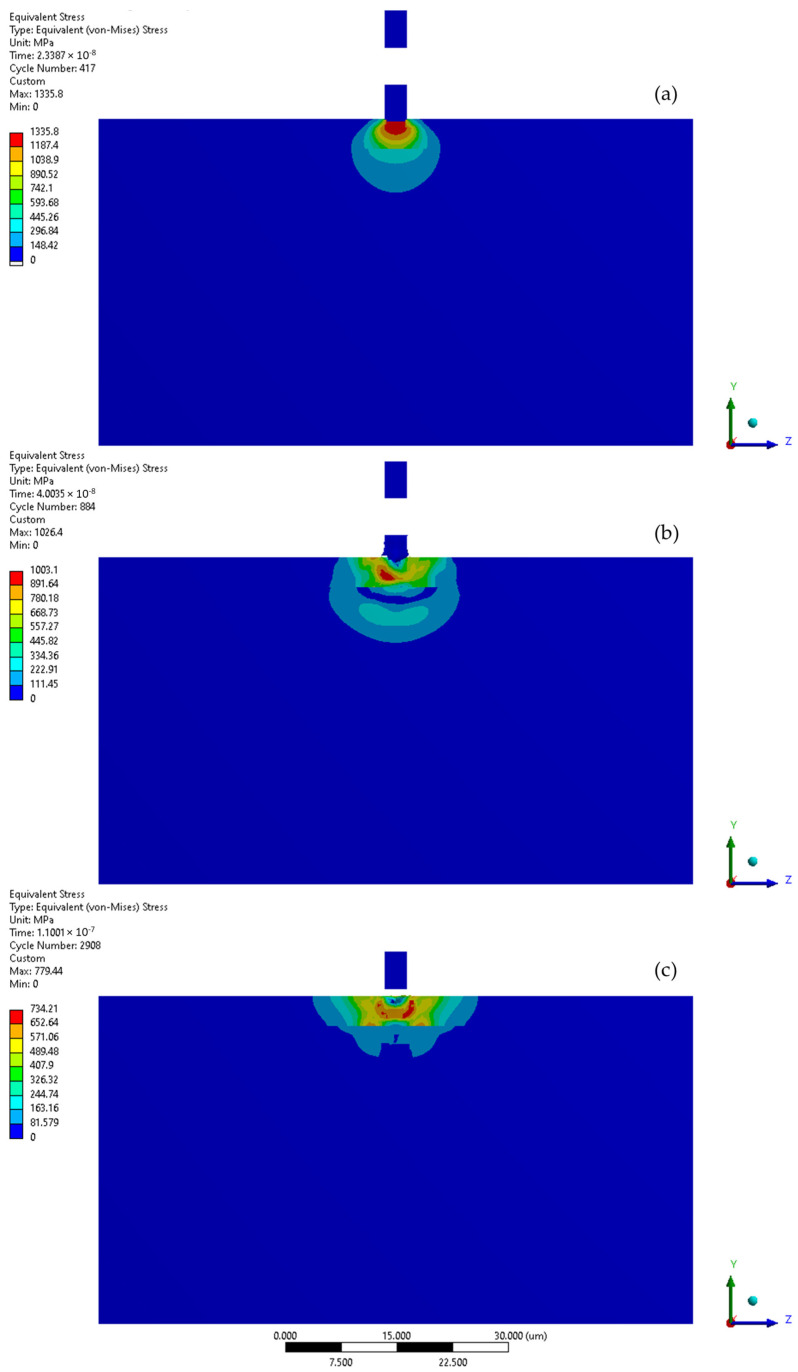
Von-Mises stress distribution throughout first microjet collision at 100 m/s: (**a**) initial stage of the collision; (**b**) state during the collision; (**c**) residual stress after the collision.

The simulation of the microjet collision at a velocity of 100 m/s generates the maximum stress of 1336 MPa in the CrN coating that forms during the initial stage (Figure 8 and Figure 9a). The elastoplastic shock wave caused by the collision was developing. The discontinuity visible in the 296.84–445.26 MPa stress contour marks the boundary between the substrate and the coating. In the second stage of collision (Figure 9b), the resulting stress was limited to 1003 MPa. The stress distribution became less uniform compared to the initial stage, resulting in a localised zone with reduced stresses. It can be expected that, following the collision, the stress levels will decrease over time, as predicted by Formulas (1) and (2). After the collision (Figure 9c), the calculated maximum residual stress was 734 MPa and the area affected by the increased stress was nearly 8 µm in diameter. Consequently, a crater with a depth of 0.5 µm was created. The Y-direction deformation range exceeds the crater depth, reaching 1.3 µm (Figure 8).

**Figure 10:**
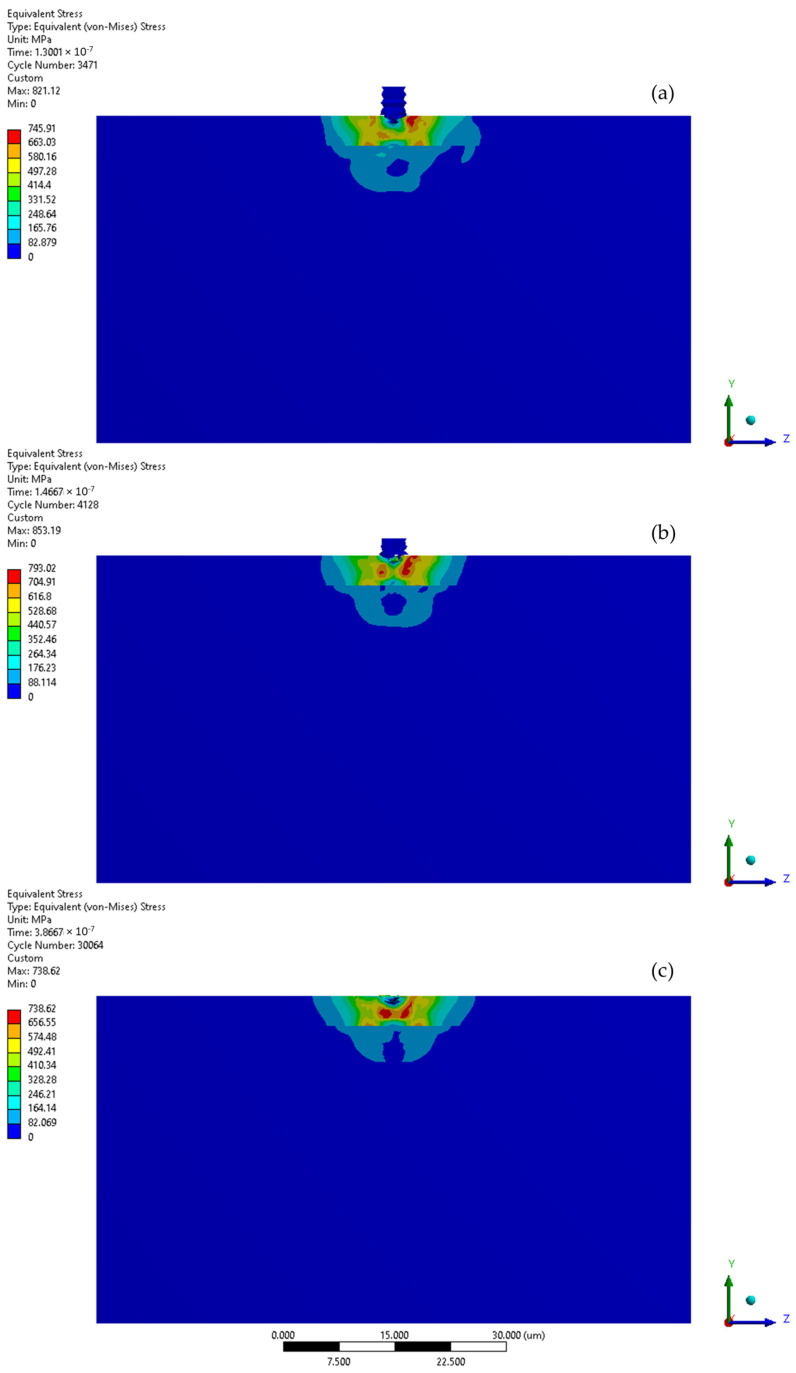
Von-Mises stress distribution throughout second microjet collision at 100 m/s: (**a**) initial stage of the collision; (**b**) state during the collision; (**c**) residual stress after the collision.

The initial stage of the subsequent microjet collision at 100 m/s creates the developed maximum stress of 746 MPa (Figure 10a). In the second stage of the subsequent collision, the maximum stress was momentarily increased to 793 MPa (Figure 10b). After the subsequent impact (Figure 10c), the peak residual stress reached 739 MPa and the area exhibiting elevated stress remained at approximately 8 µm. The reduction in residual stress observed after the subsequent collision can be described by the Hertzian contact theory [35], as the interaction between the second microjet and the sample changed from a flat to a spherical type. As an effect of subsequent collision, the depth of the crater deepened to 0.8 µm. Here, the Y-direction deformation range significantly exceeds the crater depth, reaching 2.7 µm (Figure 8).

A comparison to the degradation observed in 316L steel without coating under a 100 m/s microjet impact revealed that, although the crater depth after the second collision was similar (0.8 vs. 0.7 µm), the stress values in the CrN coating were over 2.5 times higher [25] and the protective properties of the coating prevented the formation of the exceeded yield stress zones in the substrate.

### 3.2. Microjet Collision at 200 m/s

**Figure 11:**
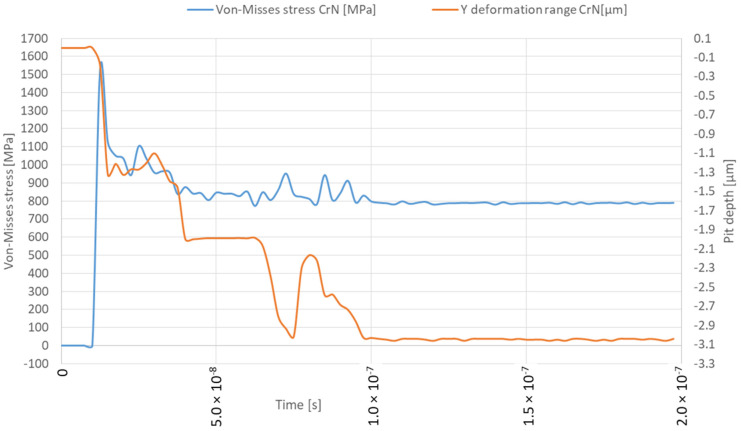
Von-Mises stresses and deformation range in the Y direction during first and second microjet collision at 200 m/s with and without protective CrN coating.

**Figure 12:**
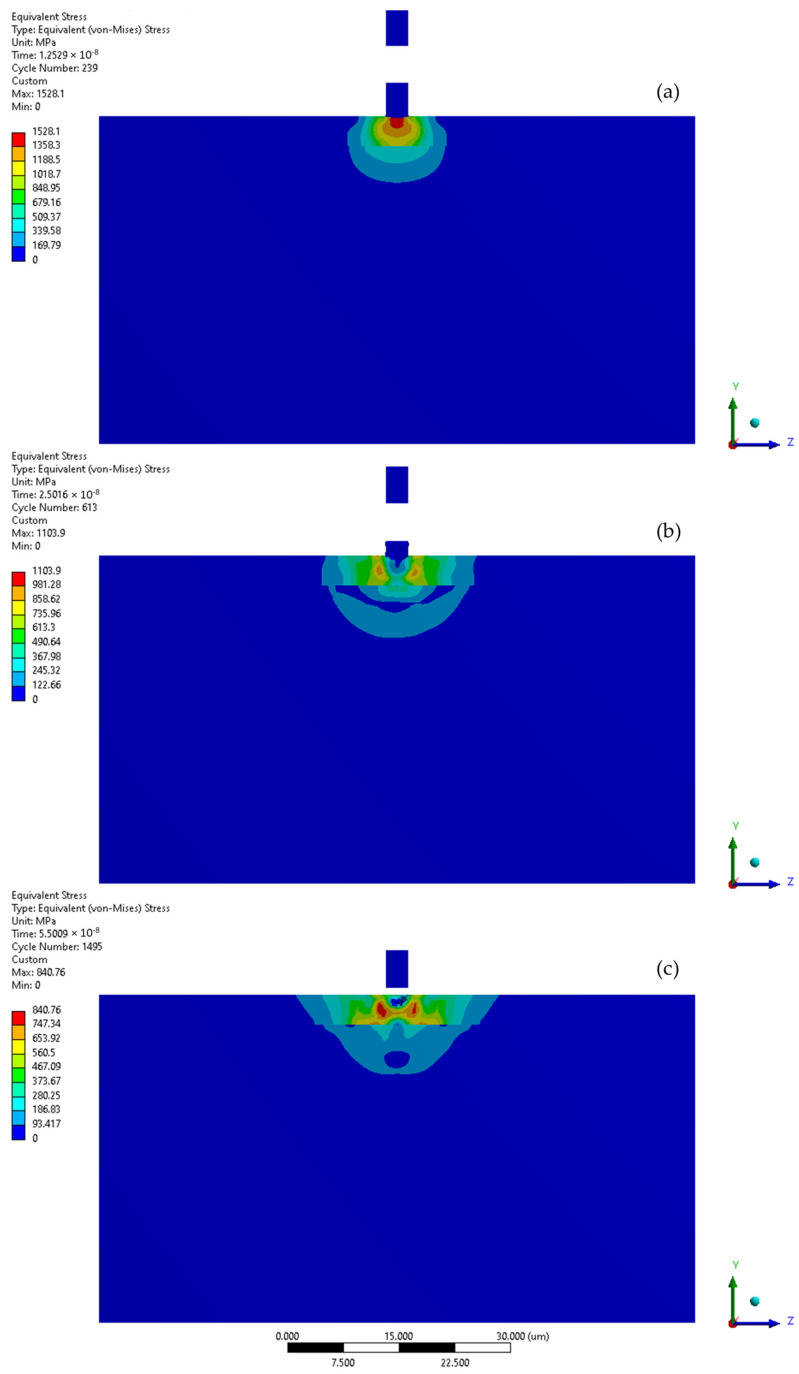
Von-Mises stress distribution throughout first microjet collision at 200 m/s: (**a**) initial stage of the collision; (**b**) state during the collision; (**c**) residual stress after the collision.

When the collision velocity is raised to 200 m/s, the peak stress during the initial stage increases to 1528 MPa (Figure 11 and Figure 12a). During the second stage of collision, the peak stress decreased to 1104 MPa (Figure 12b). The stress distribution remained almost uniform as in the initial stage, with the exception of a localised zone where the lowered stress occurs. After the impact (Figure 12c), the depth of the remaining crater was 1.2 µm, the peak residual stress was 841 MPa and the zone with increased stress was approx. 9 µm in diameter. The Y-direction deformation range significantly exceeds the crater depth, reaching 2.0 µm (Figure 11).

**Figure 13:**
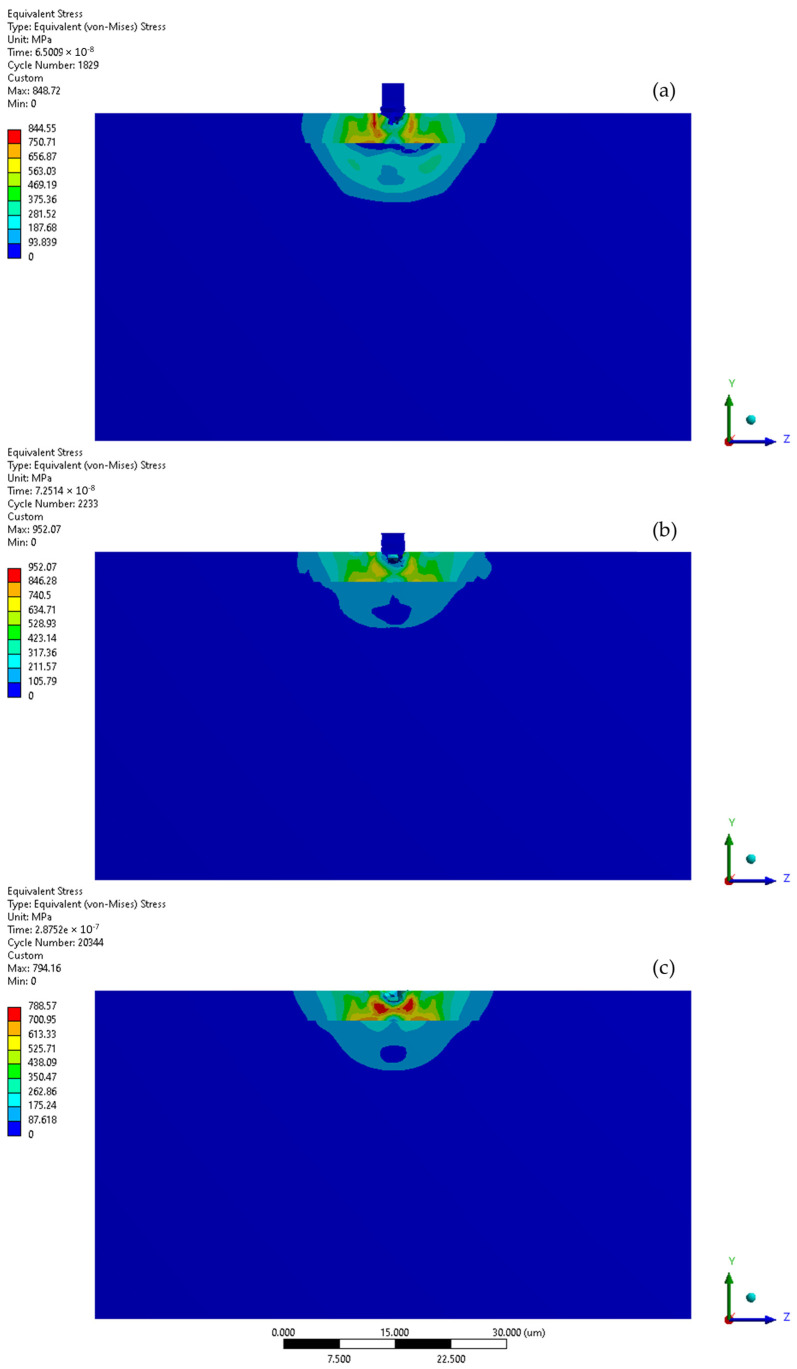
Von-Mises stress distribution throughout second microjet collision at 200 m/s: (**a**) initial stage of the collision; (**b**) state during the collision; (**c**) residual stress after the collision.

The subsequent microjet collision at 200 m/s generates a stress of 845 MPa, which further evolves during the second impact stage (Figure 13a). The maximum value of stress was reached momentarily—952 MPa (Figure 13b). The stress distribution was more uniform than in the initial stage. After the collision (Figure 13c), the peak residual stress reached 789 MPa, with the region of elevated stress extending roughly 10 µm. As a result of the subsequent collision, a 1.5 µm crater was formed. Here, the Y-direction deformation range significantly exceeds the crater depth, reaching 3.1 µm (Figure 11). Again, the exceeded yield stress of the 316L stainless steel substrate—307 MPa—was not reached after the first microjet nor the subsequent microjet collision at 200 m/s. The coating was sufficient to prevent the substrate from deforming. Again, comparison to the corresponding calculation results for the microjet impact at 200 m/s without coating [25] revealed that, although the crater depth after the second collision was slightly higher (1.5 vs. 1.3 µm) due to the protective properties of the coating, the exceeded yield stress zones in the substrate did not occur.

### 3.3. Microjet Collision at 500 m/s

**Figure 14:**
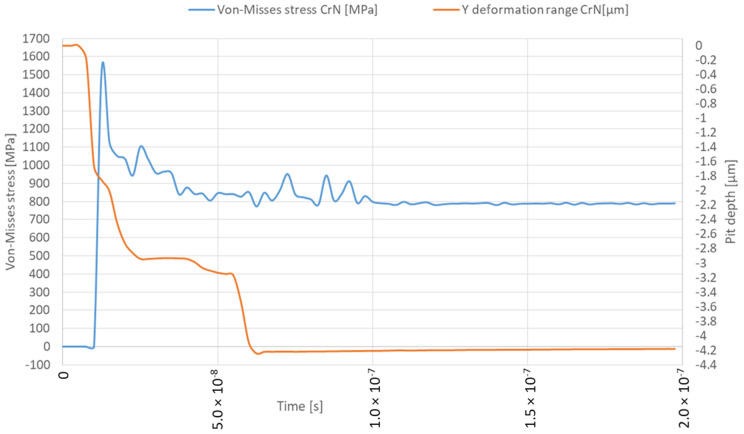
Von-Mises stresses and deformation range in the Y direction during first and second microjet collision at 500 m/s with and without protective CrN coating.

**Figure 15:**
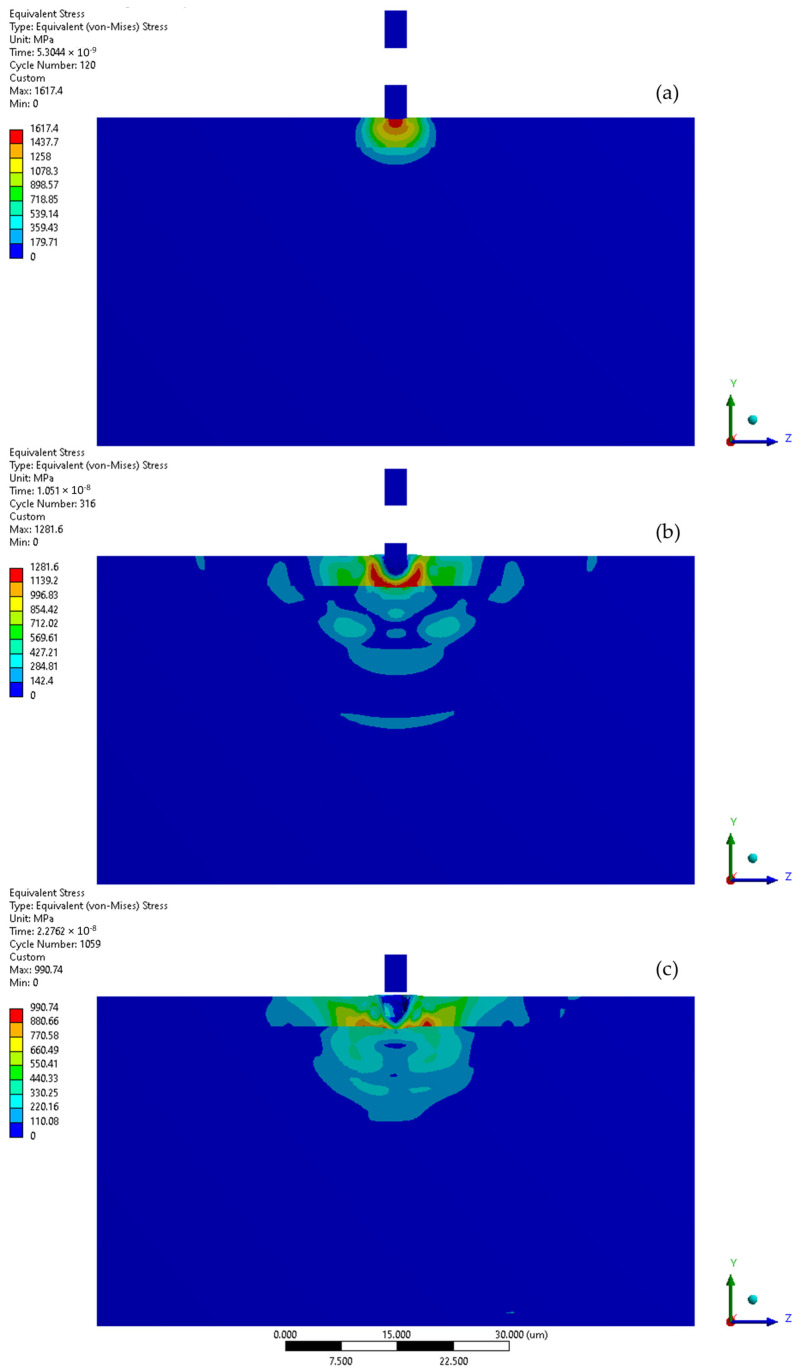
Von-Mises stress distribution throughout first microjet collision at 500 m/s: (**a**) initial stage of the collision; (**b**) state during the collision; (**c**) residual stress after the collision.

When the collision velocity is increased to 500 m/s, the maximum stress at the moment of the initial collision rises to 1617 MPa (Figure 14 and Figure 15a). In the second stage of the impact, the maximum stress decreased to 1282 MPa, triggering the FE degradation process and virtually symmetric stress distribution (Figure 15b). In this snapshot, the developing impact shockwave was also visible. Following the collision (Figure 15c), the peak calculated residual stress was 991 MPa and the area affected by this elevated stress had a diameter of approximately 11 µm. The first collision resulted in the formation of a crater with a depth of 3.5 µm. The indicated Y-direction deformation range is lower than the crater depth, reaching 3.1 µm, as the solver lost track of the element that was deactivated after reaching the failure criterion (Figure 14). Although the coating was not fully penetrated, the maximum yield stress of 307 MPa was reached in the substrate material.

**Figure 16:**
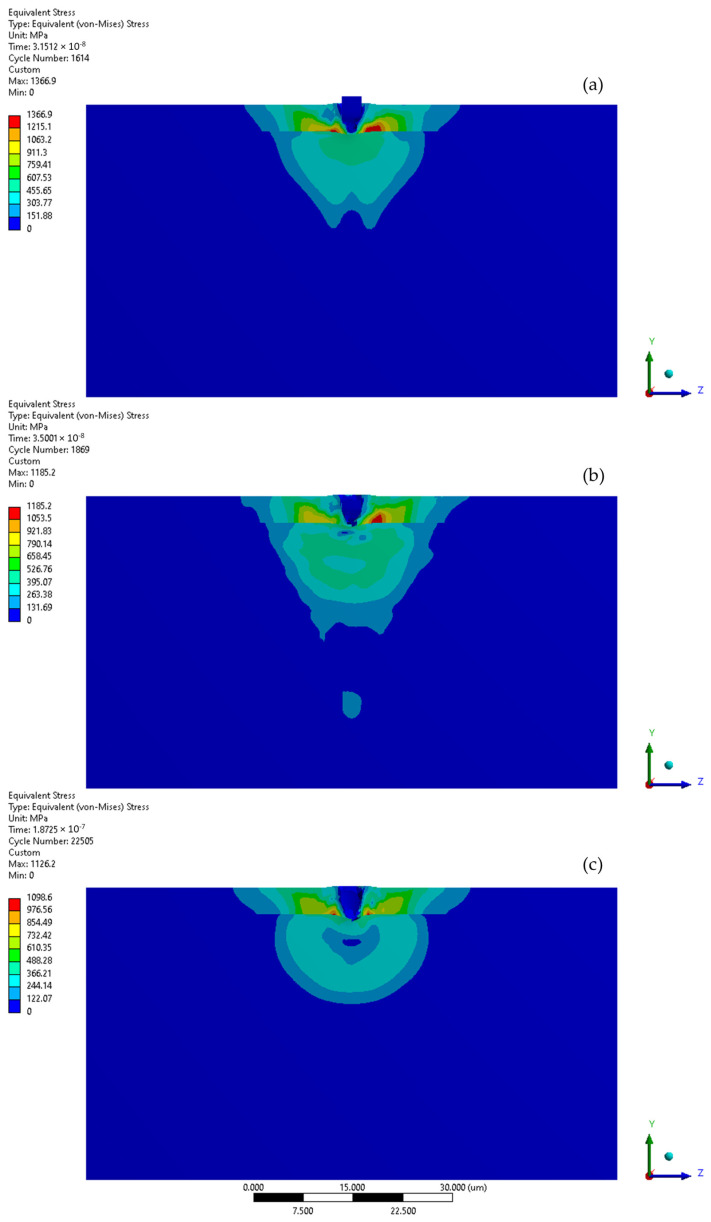
Von-Mises stress distribution throughout second microjet collision at 500 m/s: (**a**) initial stage of the collision; (**b**) state during the collision; (**c**) residual stress after the collision.

The initial stage of the subsequent microjet collision at 500 m/s generates a localised stress impulse of 1367 MPa, which leads to further finite element degradation (Figure 16a). During the second stage of the collision, the stress level dropped to 1185 MPa (Figure 16b). The asymmetry in the stress distribution visible in the initial stage increased. Following the subsequent collision (Figure 16c), the maximum residual stress reached 1099 MPa, with the area of elevated stress extending roughly 14 µm. The subsequent collision caused the crater depth to surpass 4 µm, penetrating the protective coating. Here, the indicated Y-direction deformation range is equal to the crater depth, reaching 4.2 µm (Figure 14).

**Figure 17:**
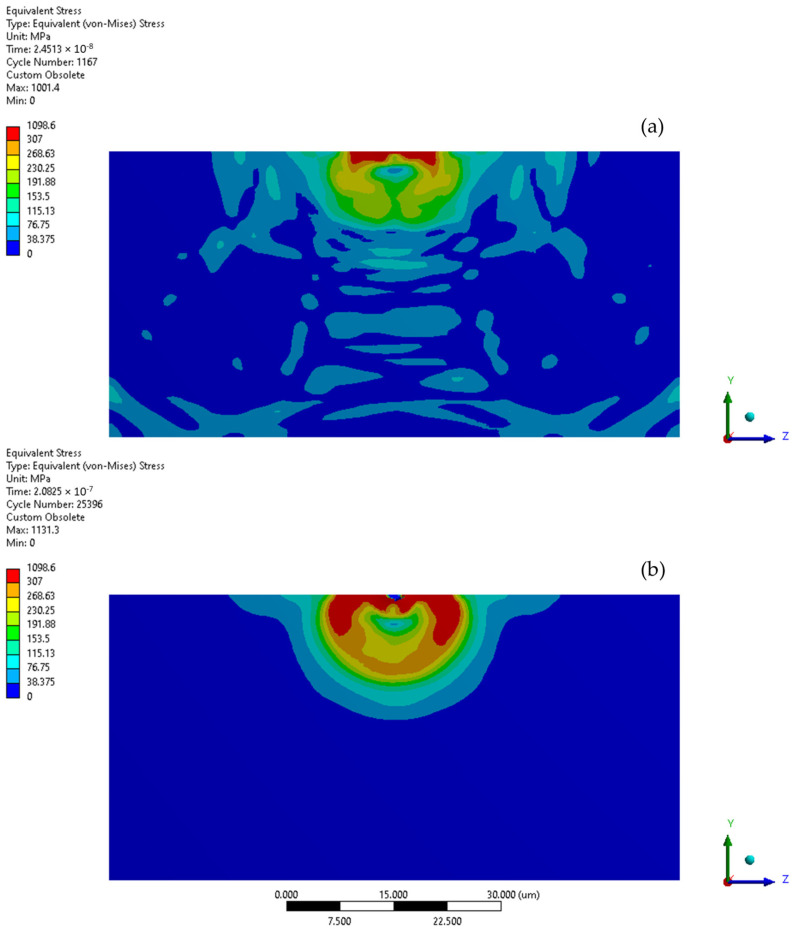
Yield stress zone exceeding 307 MPa (red) of 316L stainless steel substrate: (**a**) after first microjet collision; (**b**) after second microjet collision; at 500 m/s (coating visibility turned off—stress colour map rescaled).

Figure 17 illustrates the comparison of zones where yield stress is exceeded (indicated in red) following the initial and subsequent collision of the microjet. Elevated material stress in austenitic steel has the potential to trigger a phase transformation [36] and possibly cause local coating delamination. The region with surpassed yield stress was considerably larger following the subsequent collision as the protective effect of the coating no longer applies.

## 4. Discussion

CrN coatings are known for their high hardness and wear resistance, properties typically associated with brittleness. This means that while the coating can resist deformation and protect the underlying steel from abrasion, it may crack or chip under significant mechanical stress. Although CrN coatings themselves are not ductile, the underlying 316L steel substrate is highly ductile. Therefore, the overall performance of the coated component is influenced by the properties of both the coating and the substrate. The ductile 316L substrate can absorb and redistribute stresses to some extent, which helps mitigate the brittleness of the monolayer CrN coating.

At the point of the initial interaction between the microjet and the coating, the generated contact stress was the largest. The asymmetries of stress distribution maps observed in various stages of the collisions result from the implemented FE degradation mechanism. Performing calculations for a single microjet water hammer with collision velocities between 100 and 500 m/s revealed that the depth of the impact craters in the CrN coating varied between 0.5 µm and 3.5 µm (subsequent collision at 500 m/s exceeded 4 µm). The crater diameter ranged between 3 µm and 4 µm and the diameter of the zone of elevated stress was between 8 µm and 14 µm. Without protective coating, these deformations were slightly larger—the 316L received impact crater depths ranging from 0.7 µm to 3.6 µm (subsequent collision at 500 m/s reached 4.5 µm). The crater diameter ranged between 4.2 µm and 4.6 µm and the diameter of the zone of elevated stress was from 9 µm to 16 µm. Although the remaining damage geometries are similar, in the case of the CrN coating, the extent and influence of the increased stress zone are much shallower, mostly limited to the surface. In summary, the CrN coating provides better control over the depth and extent of impact damage with a more localised stress impact area compared to the uncoated 316L substrate. This implies that the CrN coating improves surface durability and limits the propagation of stress-induced damage.

The protective properties of the coating were effective up to collision velocities of 500 m/s. The formation of craters is driven by material deformation or degradation, which is influenced by the levels of equivalent stress and the extent of elastoplastic hardening. The thickness and microstructure of the monolayer CrN coating also influence its mechanical properties. Thicker coatings might exhibit more pronounced brittleness, while microstructural features like grain size and the presence of defects can impact performance.

At a velocity of 500 m/s, the first microjet impact creates a crater of 3.6 µm. The subsequent collision of a microjet water hammer with an identical impact velocity enhances the depth of the crater and penetrates the 4 µm CrN coating. After the subsequent impact, the zone of exceeded yield stress in the 316L substrate was considerably larger. The insufficient elastoplastic hardening did not prevent further degradation of the substrate material. The calculation results acquired for the microjet collision at 500 m/s correlate with experimental results for exposures over 1000 kPa. The stress level in the substrate decreases during the subsequent impact process, as the travelling shockwaves observed after the microimpact relax the residual stress through a stress relaxation mechanism. Similar mechanisms were observed in previous studies [25].

## 5. Conclusions

The proposed numerical simulation approach provides valuable insights into the behaviour of CrN-coated 316L stainless steel under different cavitation erosion conditions. The load parameters from cavitation pit geometries, considering strain rates at high collision velocities were estimated using the dynamic explicit method. The simulations show a clear relationship between the impact velocity and the degradation of the coating. At lower velocities (100 and 200 m/s), the CrN coating effectively protects the substrate, preventing it from exceeding its yield stress. At higher velocities (500 m/s), the protective effect of the coating diminishes, leading to substrate deformation and increased crater depth. For lower collision velocities, the protective properties of the coating prevail for the first as well as for the subsequent impact. The CrN coating demonstrates significant protective properties, reducing the extent and depth of deformation compared to uncoated 316L steel.

In applications where the coated component is subject to high wear and moderate impact, ceramic coating hardness is beneficial. However, in applications involving high impact or flexural stresses, its brittleness is a potential drawback as the substrate may be subject to delamination. In the presented results, the brittleness of the ceramic coating is counterbalanced by the ductility of the metal substrate, contributing to the overall durability of the coated component. The specific application and operational conditions play a significant role in determining the suitability of considered coatings.

The proposed numerical simulation approach can be directly applied to engineering practices, offering insight for optimising the coating thickness in response to specific cavitation load intensities. This ensures that coatings can be tailored to enhance durability and performance in hydropower machinery and other applications subjected to cavitating environments. In future studies, the effects of cavitation erosion caused by multiple successive microjet impacts over a larger surface area will be investigated, as this scenario more accurately represents the complexity of the real erosion process.

## Figures and Tables

**Table 1:** Chemical composition [%] of steel 316L substrate.

C	Mn	Si	Ni	Ti	Cr	P	S
0.01	1.79	0.53	9.56	0.15	17.05	0.025	0.027

**Table 2:** Mesh independence analysis results.

Element Size (μm)	Number of Elements	Max. Stress (MPa)	Max. Y Deformation (μm)	Run Time (h)
0.8	455,312	1258	−2.51	3
0.7	674,256	1289	−2.57	4.5
0.6	1,090,955	1336	−2.64	7
0.5	1,876,495	1343	−2.65	13
0.4	3,546,584	1347	−2.66	25

**Table 3:** The material properties of 316L steel and CrN coating adopted in numerical calculations.

Property	316L	CrN	Unit
Density	7990	5900	kg/m^3^
Young’s Modulus	193	200	GPa
Poisson’s Ratio	0.3	0.2	-
Bulk Modulus	160.8	111.1	GPa
Shear Modulus	74.2	83.3	GPa
Specific Heat	452	477	J/kgK
Strain Rate Correction	First-Order	First-Order	-
Initial Yield Stress	307	950	MPa
Hardening Exponent	0.36	0.095	MPa
Hardening Constant	275	1000	-
Strain Rate Constant	0.022	0.012	-
Thermal Softening Exponent	1	1	-
Melting Temperature	1537.9	1436.9	°C
Refresh Strain Rate	1	1	1/s
Maximum Yield Stress	600	2200	MPa

## Data Availability

The data presented in this study are available on request from the corresponding author due to the large volume of the results data set.
